# PyPedia: using the wiki paradigm as crowd sourcing environment for bioinformatics protocols

**DOI:** 10.1186/s13029-015-0042-6

**Published:** 2015-11-19

**Authors:** Alexandros Kanterakis, Joël Kuiper, George Potamias, Morris A. Swertz

**Affiliations:** University of Groningen, University Medical Center Groningen, Genomics Coordination Center, Postbus 30 001, Groningen, 9700 RB The Netherlands; Institute of Computer Science, Foundation for Research and Technology Hellas (FORTH), Nikolaou Plastira 100, Heraklion, 71110 Greece

**Keywords:** Wiki, Web services, Open science, Crowdsourcing, Python

## Abstract

**Background:**

Today researchers can choose from many bioinformatics protocols for all types of life sciences research, computational environments and coding languages. Although the majority of these are open source, few of them possess all virtues to maximize reuse and promote reproducible science. Wikipedia has proven a great tool to disseminate information and enhance collaboration between users with varying expertise and background to author qualitative content via crowdsourcing. However, it remains an open question whether the wiki paradigm can be applied to bioinformatics protocols.

**Results:**

We piloted PyPedia, a wiki where each article is both implementation and documentation of a bioinformatics computational protocol in the python language. Hyperlinks within the wiki can be used to compose complex workflows and induce reuse. A RESTful API enables code execution outside the wiki. Initial content of PyPedia contains articles for population statistics, bioinformatics format conversions and genotype imputation. Use of the easy to learn wiki syntax effectively lowers the barriers to bring expert programmers and less computer savvy researchers on the same page.

**Conclusions:**

PyPedia demonstrates how wiki can provide a collaborative development, sharing and even execution environment for biologists and bioinformaticians that complement existing resources, useful for local and multi-center research teams.

**Availability:**

PyPedia is available online at: http://www.pypedia.com. The source code and installation instructions are available at: https://github.com/kantale/PyPedia_server. The PyPedia python library is available at: https://github.com/kantale/pypedia. PyPedia is open-source, available under the BSD 2-Clause License.

**Electronic supplementary material:**

The online version of this article (doi:10.1186/s13029-015-0042-6) contains supplementary material, which is available to authorized users.

## Background

It is a general consensus that modern bioinformatics software should be useful in a community broader than the original developers. To make this possible, this software should possess certain qualitative characteristics such as performance [1], openness [2], intuitive user interaction [3] code readability and validity [4]. Developing software while keeping in accordance with all these characteristics is a tedious and resourceful process for most developers. As a consequence, many bioinformatics tools are developed in isolation to solve local or project problems without the needs of a broader community in mind. This is understandable as in academia, the developers are usually trainees that may have deep biological or statistical expertise but often lack experience of modern software management methods and development and are under pressure to deliver in a short time frame without much reward for long-term investments such as user guides, examples and unit test [5]. However, this greatly hinders synergism between bioinformaticians with similar projects in labs, institutes and multi-center consortia. So while today most software is open source and widely available, the overhead of installing, learning, configuring and validating an external bioinformatics tool for a particular type of analysis is still a major challenge and we are still far away from the vision of not only open and accessible but, more significantly, explicit, maintainable and ready to use, bioinformatics protocols [4].

Through these realizations it becomes evident that we need an environment that can guide bioinformaticians, regardless of their level, background, expertise, and programming skills, to collaborate into writing, documenting, reviewing, testing, executing, sharing and in general co-existing into the experience of biology related software development Several environments for coders exist, such as cloud9 [6] or github.com, but their technical nature often limits access for biologists who only occasionally program. More accessible solutions such as IPython notebook [7, 8] come closer, but are in general addressed to experienced users, they lack a central repository of publicly editable methods and do not offer version control. Meanwhile, Wikipedia has been successful as a low-barrier environment for very diverse content providers spanning from all the spectrums of expertise and backgrounds to collaborate into creating new articles and co-develop them to high quality. The advantages of the wiki principle in the scientific content management have already been discussed [9–11] and the concept of wikis has already been used in the area of bioinformatics, such as Wikigenes [12], SNPedia [13], GeneWiki [14] and, semantic integration [15, 16]. Most relevant wiki for programming is Rosetta Code (Mol, 2007), which contains mainly a wiki of code snippets for known computational problems but not optimized for “real world problems”.

In this paper we describe PyPedia, an effort to employ the wiki concept in order to provide a crowdsourced environment where bioinformaticians can share their expertise and create or edit qualitative methods in the python language. Moreover, users can experiment online with various methods and perform basic interactive data analysis. Finally PyPedia can act as a simple python library for a variety of bioinformatics methods.

## Implementation

PyPedia is a wiki based on MediaWiki, the wiki engine that powers Wikipedia. As in Wikipedia, the content is divided in articles. In PyPedia each article is either a python function or a python class. The title of each article has the same name as the function/class that it contains. In Wikipedia, we can place a link to any other article with a simple notation (also called wikilink, or internal link). Similarly in PyPedia a function call or a class instantiation is automatically a wikilink to the called/instantiated function/class. Moreover, this wikilink, functionally connects an article with the linked article as a programming dependency. For example when the function ‘PLD’ (short for Pairwise Linkage Disequilibrium) calls the function ‘MAF’ (short for Minor Allele Frequency) then the function ‘MAF’ becomes automatically a wikilink in the article ‘PLD’ that point to ‘MAF’. When a user executes the ‘PLD’ method, then the code that is also in the ‘MAF’ article is also executed (when called by ‘PLD’). The user does not have to make any special ‘import’ statement since this is taken care by PyPedia. By implementing this, we have converted a wiki engine to a python library that can grow multidimensional while users add more articles. Users can request to download the code for the ‘PLD’ function that will also contain recursively all the dependencies hosted in PyPedia. In the remaining of this chapter we detail the functionality that allows different ways of sharing, execution and testing of the code, quality control and protection from malevolent edits.

### Python

For this pilot we decided to use Python because its design philosophy emphasizes in code readability while having remarkable power. It features a readable syntax, functional and object-oriented abilities, exception handling, high level data types and dynamic typing. It offers implementations in all common computer architectures and operating systems and most importantly a huge variety of ready-to-use packages for common programming tasks. It is between the most popular scripting programming languages and has a dominant position in the area of bioinformatics. E.g., BioPython [17] is the most known library for molecular biology and bioinformatics whereas PyCogent [18] focuses in sequence management and genomic biology. Other libraries include DendroPy [19] for phylogenetic computing, Biskit [20] for structural bioinformatics, pymzML [21] for mass spectrometry data and Pybedtools [22], Pyicos [23] for sequencing. These tools can be combined with more generic libraries for scientific computing like scipy [24] for numerical analysis and matplotlib [25] for plotting. PyPedia can act as a community maintained glue library between these packages by enriching their abilities, providing conversion functions and demonstrating common use cases.

### Wiki

PyPedia is an extension to the Mediawiki content management system mostly known as the backend of the Wikipedia project. Mediawiki is a modern Content Management System with many features like versioning, edit tracking, indexing/querying, rich content (for example LaTeX math formatting), templates and multiple user groups. Moreover, Mediawiki is highly extensible since it supports connections with external software that can alter its standard behavior. These connections are called hooks. PyPedia’s extensions to Mediawiki consist of two hooks. The first hook is activated when a new article is created and inserts the initial content that predefines the structure of the article. The second hook is activated when a user submits new content and performs checks to verify the validity of the edit.

**Fig. 1:**
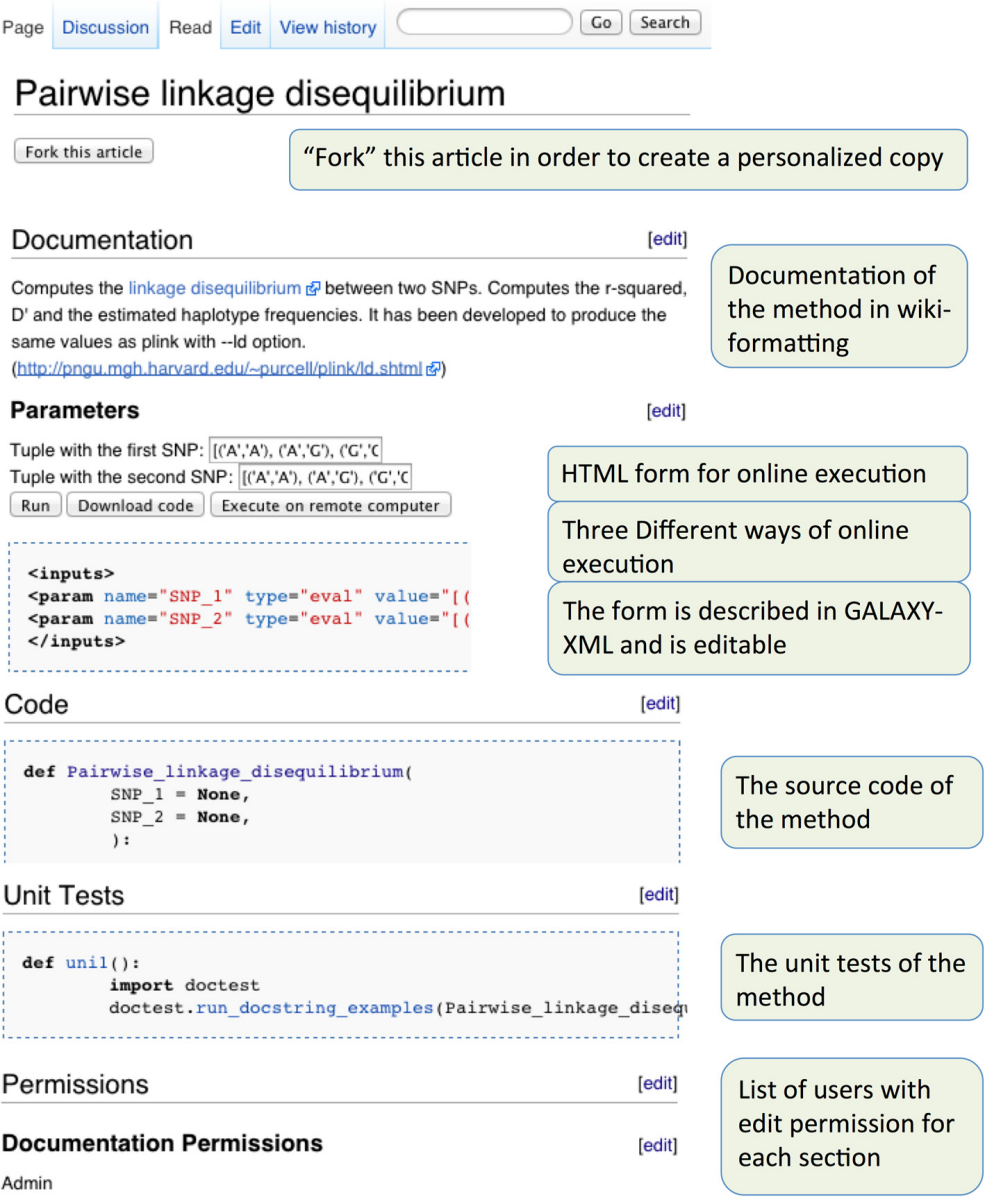
The structure of an article in PyPedia. An article has a predefined structure. The “Code”, “Development Code” (not shown) and “Unit Tests” sections contain python code. The rest sections define the documentation, parameters and permissions of the article

Each PyPedia article follows a predefined structure whereas addition or deletion of sections is not allowed in order to preserve uniformity over all methods. Along with the source code, each article has sections that provide documentation, user parameters, under development code, unit tests and edit permissions of the method (Fig. 1). In the following paragraphs we explain the use of each section and the checks that are applied.

**Fig. 2:**
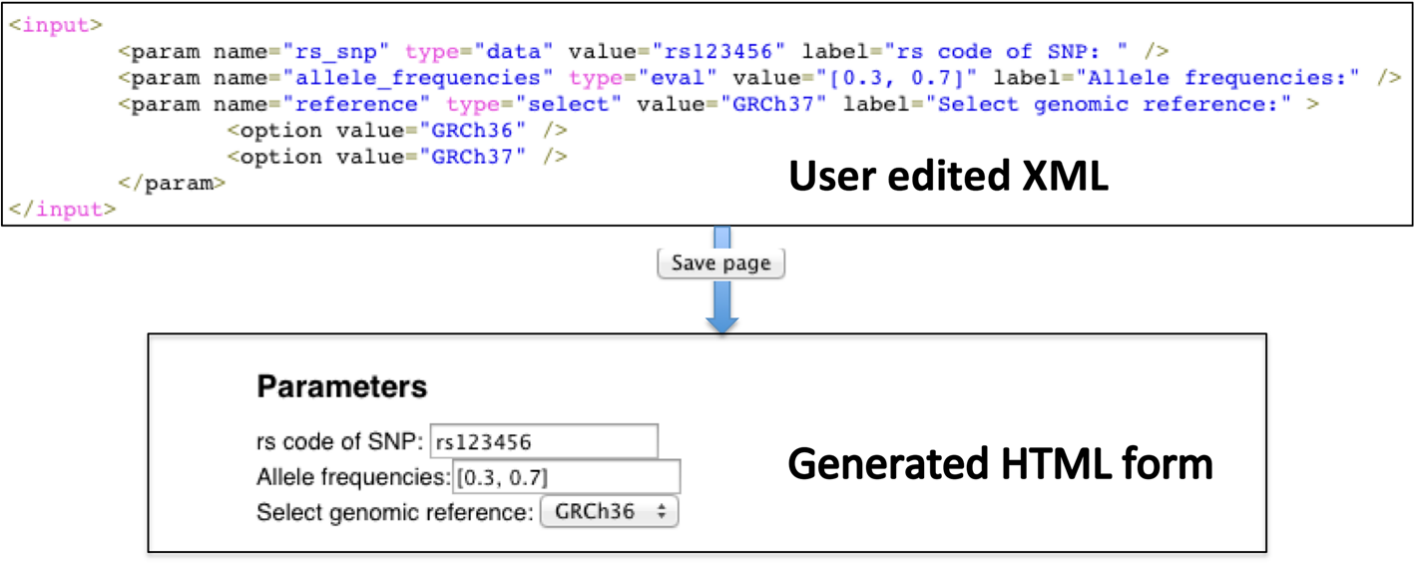
Creating parameters. An example of generating a parameters form. The user defines the parameters in Galaxy XML (upper part) and upon saving it is converted to an HTML form

The first section is the “Documentation”. In this section the user documents the method, explains the parameters, provides references and in general contributes with any information that will aim the potential user to use this method. The documentation is done with wikitext, that is a simple markup language for the visual enrichment of the provided text with HTML elements. Among others, users can assign categories, add images, tables, hyperlinks and any element supported by Mediawiki. In the “Parameters” section a user can create or edit an HTML form. This form can be used to fill-in parameters of the method before executing it. The different ways for executing the method after filling this HTML form are explained at the “Using PyPedia” paragraph. The format used for the creation of this form is a subset of the Galaxy [26] XML (Extensible Markup Language) tool configuration language and its outline is shown in (Fig. 2).

For each parameter a <*param*> XML element has to be defined. The “name” attribute of the “param” element should have the same value as a parameter of the python function that this article describes. The “type” attribute can be either “data” if the input will be treated as a simple string or “eval” if it is to be treated as a Python expression (i.e. “a” :1). Finally if the “type” attribute is “select” then a combo-box will be created. The possible options of the combo box can be defined with subsequent <*option*> elements. After a user edits and submits the parameters the second hook parses the XML and creates the HTML form that is displayed in the article’s page.

As with the “Documentation”, the “See also” section can contain arbitrary wiki markup. The difference is that this section is focused into providing inner links to similar articles, or to articles that call or are called by this method. Similarly the “Return" section should give information about the return value of this method.

The “Code" section is where the source code of the method resides. In this section a user can submit an implementation through either a python function or class. The only limitation is that the function’s (or class’) name should be identical as the article’s title. Virtually, all methods in PyPedia belong to the same namespace. This means that a simple function call (or class instantiation) is enough to load the code of another article. Since there is no need to import, we conform to the wiki philosophy where inner linking should be intuitive and simple.

The “Unit tests” section contains functions that test the validity of the code submitted in the “Code” section. Unit testing is the process of automatically triggering the invoking of methods that test the integrity of recently submitted code. It is an important component since it ensures that recent edits didn’t break existing functionality and guarantees some minimum code integrity [27]. In PyPedia unit tests are functions that take no options and return True or False whether the implemented test succeeds or not. If a unit test returns a string then it is considered that it failed and the returned text appears as an error message to the user.

When an edit in the source code or the unit tests is made the following procedure is executed before saving: The source code and the unit tests are parsed and all the referenced methods are identified and loaded recursively. The dependency-free source code is sent through an Ajax call to a python sandbox. This sandbox contains a virtual environment where the execution of python code cannot cause any side effect even if the code is deliberately malicious. In this environment we have installed Anaconda [28], which is a preconfigured version of Python with hundreds of scientific packages including BioPython. This constitutes the ideal environment for testing the user-provided non-secure code. In this environment we execute the unit tests and any violation is reported back to the user. If the execution is successful then the edit is saved. The environment for code editing is based on the ACE code editor for the web that offers syntax highlighting, auto indentation and other modern IDE (Integrated Development Environment) features. Offline editing in a local environment is also supported (Additional file 1).

Each one of the “Document”, “Code”, “Unit tests” and “Permissions” sections can have their own permissions settings. Initially, when an article is created, only the creator user is allowed to edit each one of these sections. By editing the “Permissions” section the user can declare in a comma separated list additional users that are allowed to edit these sections. Special usernames include “ALL” for all (even anonymous) users and “SIGNED” for all signed in users. Although openness is always encouraged we allow user restricted article editing. This allows the creation of sub-communities where only specific users are allowed to edit some of the articles. As with all Mediawiki environments, there also exists an open “Discussion” page for each article for general comment submission.

### Using PyPedia

**Fig. 3:**
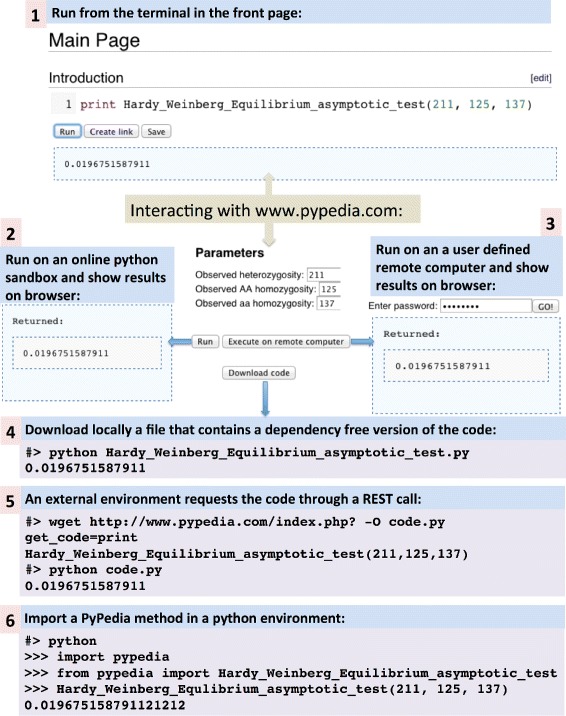
Executing code hosted in PyPedia. The six different ways of executing code hosted in PyPedia as they are described in the “Using PyPedia” section. Methods 1,2,3,4 require interaction with www.pypedia.com. Method 5 is through the RESTful interface and method 6 is through the python pypedia package

There are six different ways to perform an analysis with code hosted in PyPedia. Four of them are by directly interacting with the pypedia.com site, one with the pypedia python library and one with a RESTful interface (see Fig. 3). In the remaining of this chapter we will describe these methods.

#### From the front-page text editor

In the front page of pypedia.com exists a text editor implemented in JavaScript, called CodeMirror. It emulates an interactive python environment where users can experiment and develop custom solutions. A user can insert python code that includes calls to PyPedia functions and classes. By pressing the ‘Run’ button, the code is parsed and the dependency-free code is formed. This code is submitted through an Ajax call to the python sandbox. The results are asynchronously transmitted back and shown to the article’s page as soon as the execution finishes. Apart from simple text the results can also be graphs or any arbitrary HTML element. The analysis command can be converted to a URL with the ‘Create Link’ button in the front page. Thus sharing the complete analysis is easy as sending a URL.

The next three methods require interaction with a specific article’s page. As it has been described before, each article contains a “Parameters” section. This section contains an editable HTML form. A user can fill this form with values that act as parameters to the function that this article contains. It is important to note that for these execution methods no knowledge of python language or programming is required. As with any website that contains a bioinformatics service, a user, only has to fill in the parameters in order to execute a method. There are three ways to execute this function with the filled-in values:

#### By pressing the ‘Run’ button

Similarly to above, with this button the dependency free code is submitted to the python sandbox and the results are shown on the browser.

#### By pressing the ‘Download code’ button

In that case the dependency-free code is downloaded in a file that has the same name as the title of the article. This file can then run in an Anaconda python environment.

#### By pressing the ‘Execute on remote computer’ button

A user can execute the dependency-free code in a remote computer of her choice. To do that, the user initially has to declare the specifications of the remote computer in her user’s page. The user page is a special set of articles where editors can create a personal profile. In this page, users can create a section titled “ssh” and then fill in the hostname, username and execution path of a remote computer. For example:



The Mediawiki database schema has been altered in order to store these elements in a separate table and its contents are never shown in any page. Once these elements are stored a user can execute the dependency-free code in this remote computer by pressing the button “Execute on remote computer” in any article. Then, a password prompt appears in the page and after completing it, PyPedia maintains a SSH connection to the declared remote computer, executes the code and fetches the results in a new browser tab. The results contain the method’s output, returned values and potential errors. This execution method streamlines the procedure between setting up an execution environment and the process of installing, configuring and executing the desired software. Tools that utilize collaborative data analysis (i.e. GaggleBridge [29]) can benefit from this approach. A simple and common example is when a group of researchers need to share a computational environment (i.e. in Amazon EC2) in order to perform a common bioinformatics task.

#### Via the RESTful API

The RESTful web service has the following specification:



With this request, any user or external tool can receive the dependency-free code. One important parameter of the RESTful API (Application Programming Interface) is the “b_timestamp” (b stands for ‘before’). With this parameter we can request a specific ‘frozen’ version of the code. When it is defined the API returns the most recent version of the code that was edited before the declared timestamp. This parameter is applied recursively for all the articles that the API requests code from. By defining this parameter we can ensure that the returned code will always be the same regardless the edits that may have happened after a specific edit and may have changed the method’s functionality. Sharing a link with the “get_code” and “b_timestamp” parameters guarantees reproducibility of the performed analysis.

It is also possible to execute code via the RESTful API. This execution is bounded by the limited time and memory resources of the sandbox. To execute a code:



#### With the PyPedia python library

Through this library, a user can download the code of a PyPedia article directly to a local Python namespace. For example assuming a Python version 2.7 or higher environment, a user types:



This import maintains an HTTP connection between a local environment and the pypedia.com website. From that point on, an import of a PyPedia function is easy as:



With this command, the code of the “Pairwise_linkage_disequilibrium” article in www.pypedia.com, is downloaded, compiled and loaded into the current namespace. Function updates are available for downloading and invoking as soon as a user submits them to the wiki. The invoking of the function is a python function call. For example to assess the pairwise linkage disequilibrium of two SNPs (Single-Nucleotide Polymorphism) genotyped in four individuals with respective genotypes AA, AG, GG, GA and AA, AG, GG, AA the command is:



The semantics of the returned values are explained in the “Documentation” section of the method’s article. This documentation is part of the downloaded function as a python’s documentation string and can be accessed by calling the __doc__ member of the function. For example:



Additional features of this library include cached downloads and debug information. The complete documentation is available at PyPedia web site and in Additional file 1. The python library is available at: https://github.com/kantale/pypedia.

### Quality control

One of the main dangers of crowdsourced management systems is the deliberate (or accidental) import of malicious code. To compensate this, the articles are split into two namespaces: (1) the default “User” namespace that contains unsafe, arbitrary submitted from any signed in user and (2) the “Validated” namespace that contains validated, qualitative and safe code approved by the administrators. The distinction between these is that the User namespace has the suffix _user_ <*username*> on the article’s name. Articles from the “Validated” namespace do note contain links to articles in the “User” namespaces. Moreover execution of articles in the “User” namespaces is allowed only in the python sandbox and never in the user’s environment. Additional file 2 contains more details regarding this distinction.

## Results

We have been using PyPedia for several years as an ongoing experiment to validate its use. As with any wiki, the content of PyPedia is constantly increasing since new methods are added and revised. In this paragraph we evaluate PyPedia by demonstrating how the current content can be used to address some common bioinformatics tasks. In Additional file 3 we present an analysis scenario that includes most of the methods of this paragraph. All available methods that belong in the Validated category can be accessed in the following link: http://www.pypedia.com/index.php/Category:Validated.

### Use case 1: Basic genomic statistics

In the area of genomics statistics, PyPedia contains methods for the estimation of a SNP’s minor allele frequency and Hardy Weinberg Equilibrium statistic. For the later, two methods are available, the exact test [30] and the asymptotic test [31]. Also as we have demonstrated PyPedia offers a method for the estimation of linkage disequilibrium between two SNPs. It also contains methods for allelic and genotypic association tests and trend tests of association between disease and markers. These methods have been validated to produce identical values with the well known PLINK software [32]. Although PLINK and similar tools are of high quality and extensively tested, they are mostly used as a black box by bioinformaticians. Given the rise of programming courses in biology curricula, approaches like PyPedia that import qualitative and community maintained methods in programming environments, allow for higher flexibility, transparency and versatility on the performed analysis.

### Use case 2: Format convertors

Format conversion is a common, usually tedious and error-prone bioinformatics tasks. There are very few formats that have been universally established as standards and it is very common phenomenon for a new bioinformatics tool to introduce a new format. The majority of bioinformatics formats are tab delimited text files where although the conversion does not require any sophisticated programming work, it consumes considerable time for researchers to understand the semantics and to make sure that no information is lost during the conversion. Consequently this process hinders the collaboration among researchers and impedes the integration of bioinformatics tools. We used PyPedia to collect and share a set of of “readers” and “writers” for a variety of known formats. These formats are: PLINK’s PED and MAP, PLINK’s transposed files (TPED and TFAM), BEAGLE [33], Impute2 [34], MERLIN [35] and VCF [36]. For example, “PLINK_reader()” is a method to read PLINK’s PED and MAP files. All readers are implemented as python generators. This case shows how by combining the relatively small ‘wiki pages’ with readers and writers we can routinely perform any conversion between these formats. More significantly, any user can contribute by adding a new format or refining an existing one. The method ‘bioinformatics_format_convert()’ offers a convenient wrapper for these methods.

### Use case 3: Genotype imputation

Genomic imputation [37] is a popular statistical method to enrich the set of markers of a GWAS (Genome-Wide Association Study) study with markers from a dense and large-scale population genetic experiment such as the 1000 Genomes Project [38] or the Genome of the Netherlands [39]. However, imputation involves many steps and typically needs a High Performance Computational Environment (HPCE) such as cluster or grid. We used PyPedia to define the class ‘Imputation’ that can create all necessary scripts and submit them to an HPCE, building on a class named ‘Molgenis_compute’ which is a wrapper for the Molgenis-compute [40] tool that can run scripts on a remote computer cluster. This case shows how PyPedia can glue together different complex and diverse components (not necessarily in Python). The ‘Imputation’ article contains detailed directions of how to perform genetic imputation with this class: http://www.pypedia.com/index.php/Imputation.

### Use case 4: QQ-plots

**Fig. 4:**
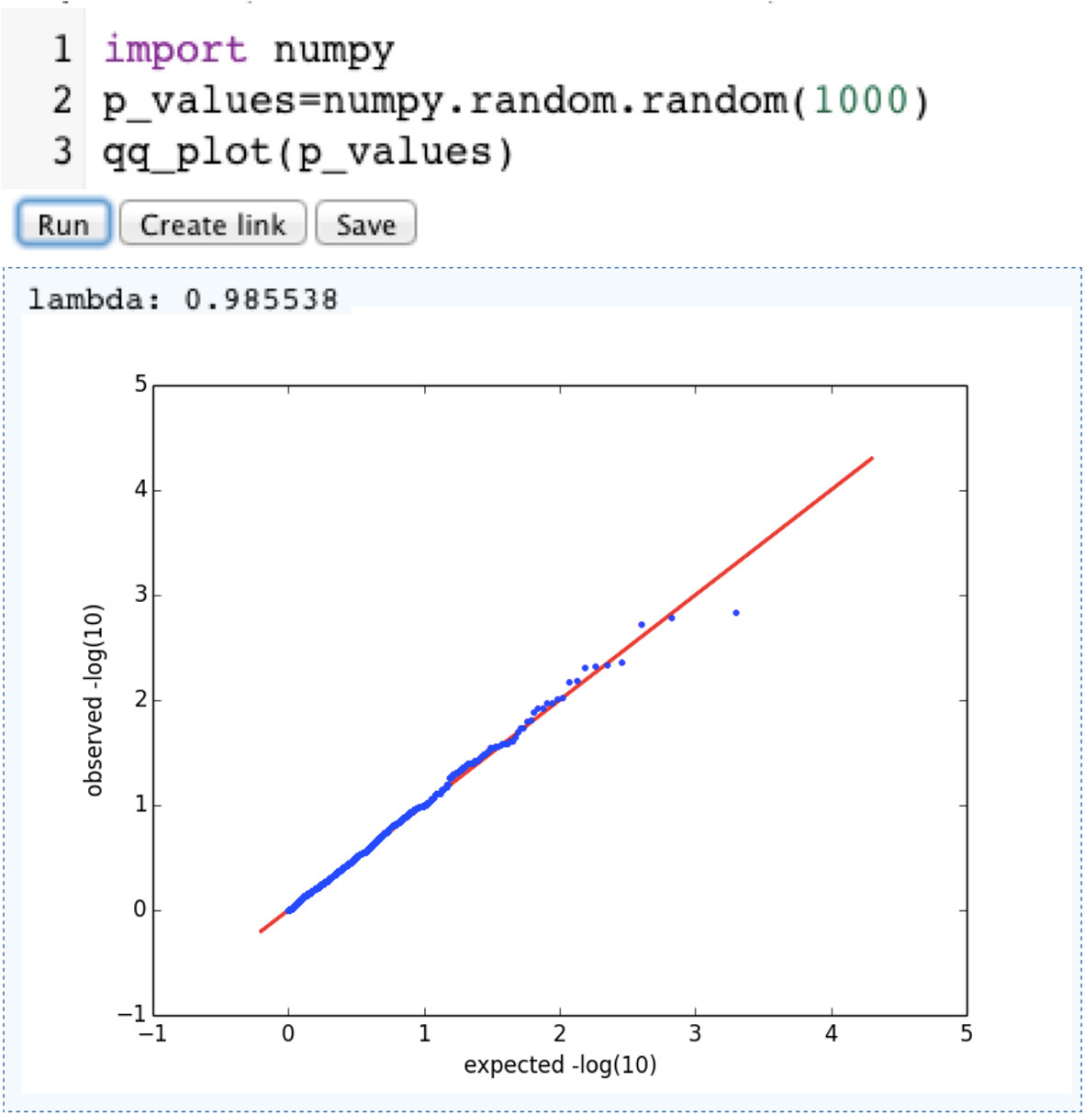
An example of a graph generated online from a PyPedia article. Graphics output can be embedded in PyPedia, such as to provide full provenance for figures in scientific publications

This is a simple use case to demonstrate the interactive generation of plots. The article qq_plot contains the code to generate quantile-quantile plots from p-values coming for example from a GWAS association testing. The plot is generated asynchronously and presented to the user as soon as it is created. This use demonstrates how also graphics producing methods can be integrated, which is ideal to store reproducible version of figures as published in papers (see Fig. 4).

### Use case 5: Reproduction of published research

In this section we demonstrate how PyPedia can be a medium for reproduction of published research. As an example we select the article from DeBoever et al. [41]. The authors of this paper have make public all the code and data required for reproducing the results and figures of the article. The code resides in a github repository (https://github.com/cdeboever3/deboever-sf3b1-2015) in the format of IPython notebooks. The data are available in the figshare website (http://figshare.com/articles/deboever_sf3b1_2014/1120663). Pypedia contains the method “notebook_runner()” which executes the entire code contained in a IPython notebook. Moreover PyPedia contains methods to download data, install external packages, decompress and manage files. To reproduce the first figure of this article, one has to run:



Since these commands take a long time and require significant disk space, they can only run in a local python environment.

To ease the procedure of configuring a pypedia environment that contains all scientific and latex libraries necessary for qualitative figure production we have created a Docker image. Docker [42] is an open-source project for creating and sharing images of operating systems that contain preconfigured environments for various solutions. By sharing a Docker image, the complete effort for installing and configuring tools and packages is eliminated. This can contribute significantly to research reproducibility [43] especially in the area of bioinformatics [44]. The pypedia Docker image is a available at https://hub.docker.com/r/kantale/pypedia/.

## Discussion

Currently PyPedia contains 354 pages (or methods) with 63 registered users. In average every page has 5.4 edits. Since the ‘fork’ feature was added recently, almost all of the pages are novel articles. PyPedia has been online for a short period of time (6 months) and additional user statistics are not available. We plan to publish user statistics after an adequate usage of the system. Furthermore, these statistics will guide us to enhance PyPedia.

### Positive aspects of the wiki paradigm

PyPedia is an effort to apply the wiki paradigm into bioinformatics methods development. The wiki paradigm can be defined as the mass and collaborative submission of unstructured information by a diverse or loosely coupled community, also called crowdsourcing [45]. Another feature is in terms of evolutionary adaptation: the content is dynamic and constantly developed as users with different abilities and perspectives edit it. Only the beneficial to the community edits stay, or “survive”, thus ensuring that most relevant articles are incrementally improved over time while irrelevant pages are removed [46]. Finally, the wiki approach can alleviate significant and constantly increasing effort and time needed to validate, maintaining and document to ease realization of the e-science vision [4] by integrally stimulate essential best practices:

**Version-control system** One of the primary characteristics of the MediaWiki is the additive model and the versioning system. All edits and the meta-information such as authors, dates and comments are stored and tracked. With the addition of the “b_timestamp” API parameter users can acquire and share a specific, time-bounded version of the code, contributing to the reproducibility of an analysis.

**Material tracking** All software, configuration steps and parameters that were used as processing steps to generate scientific results should be tracked. Additionally should be easily shared and reproducible by third parties [2]. Researchers that performed an experiment with PyPedia methods can provide links to the revisions of the articles that were used (permalinks). Any other party can use these permalinks to access the specific version of the methods and perform the same computational steps, even if the respective articles have changed since then.

**Write testable software** This principle recommends the use of small, modular components that can be easily tested and combined into larger solutions. This is the essence of the PyPedia functionality. Every article is a small independently developed and tested module. The extension undertakes seamlessly the combination of articles into integrated programs when this is needed.

**Encourage sharing of software** Unlike traditional open source policies of releasing the code under distinct versions, in PyPedia, the whole continuous process of developing is open. Moreover, the content is released under the BSD license that is one of the most open and permissive licenses that allows re-use and re-mix of the content under the condition that suitable attribution is given.

### Criticism of the wiki model

The major criticism against the use of the wiki paradigm in the scientific context is that the crowd does not always exhibit the required synergy into submitting qualitative articles [47]. Usually disagreements arise that require the intervention of an expert that is not always recognized from the whole community. There is also the impression that qualitative code is difficult to find and hence wiki curated code is of poor quality. In PyPedia, we therefore provide an optional system where the submission of alternative content for similar methods can be done through “User” articles. Any user can create a copy of an existing algorithm under her user name and submit an alternative version. This is similar to the “fork” procedure in the revision control systems. In addition we created articles in a “Validated” category that can be more closely managed by (project/lab/consortium) administrators and are updated from the pool of User articles under the strict qualitative criteria (see also Additional file 2).

Another issue of the wiki content is the deliberately malicious edits, also referred as vandalism and common spam. Vandalism is limited by explicitly setting user rights to every section of the article. So only sections that allow anonymous edits are prone to this. The level of edit-openness and thus the risk for vandalism is left to the authors of the articles although administrators can take action when they identify it. To manage spam we have adopted the CAPTCHA approach.

Yet another criticism refers to the level of maturity of the research community into adopting open source tactics [5]. Some authors are reluctant to publish code either because they think it is not good enough or because they afraid to share. Other authors are convinced that sharing does not only benefit the community that uses an open-source project but the original authors as well in terms of citations, visibility as an expert, and funding opportunities.

A final note is about reproducibility, which is one of the key aspects of the modern e-science era. It has been argued [48] that modern software infrastructure lacks mechanisms that will enable the automatic sharing and reproduction of published results and that subsequently hinders scientific advancement in general.

### Wiki versus GIT and IPython

Currently, the most prominent medium for scientific collaboration is the GIT tool [49] through the several GIT hosting services such as GitHub and BitBucket. Especially for python developers, GitHub is able to render online IPython notebooks. Moreover, PyPedia as a wiki, contains a versioning mechanism which is inferior to GIT’s relevant system. Nevertheless, the ‘wiki’ philosophy is completely absent from the GIT model. As a consequence, scientists, still have to search for methods in different repositories, find ways to combine different code bases and go through unavailable or incomplete documentation.

PyPedia, as a wiki, encourages users to contribute their code not for the purpose of just storing it in an open version control system (which is mostly the case of Github-like repositories) but to contribute in a generic project. That means that the code has to cover a generic problem, to be well written, documented, tested and more significantly to use other wiki methods. By following these principles, data analyzed or generated with PyPedia methods are easier to be interpreted. This is orthogonal to traditional data analysis in science that happens mainly with methods that even when they are well written, the justification of developing them is often omitted. Nevertheless since the majority of scientific code resides in git repositories, in our future work, we plan to shorten the distance between wiki and GIT, that is, to handle the code management with a GIT compatible service instead of MediaWiki.

Another issue is the IDE features of PyPedia. Modern IDE environments offer far superior abilities compared to the plugins of PyPedia. These IDE-like plugins of PyPedia have the purpose to aim users to apply simple changes rather than to be an adequate environment for the development of large scale solutions. Nevertheless, PyPedia can function as a modern repository of highly qualitative code with simple editing abilities.

Finally, the main usage of PyPedia is not for interactive data analysis since other tools like IPython, Python(x,y) [50] and Spyder [51] are more targeted to this purpose and have superior capabilities compared to PyPedia’s web based environment. PyPedia is designed to be complementary to these tools when it comes to interactive data analysis. That means that code hosted in PyPedia can be executed in these tools interactively and the opposite, meaning that code developed on these tools can be uploaded to PyPedia. As an example in Additional file 3 we demonstrate an interactive data analysis from code hosted in PyPedia combined with code developed locally. In contrast, code hosted in Github cannot be executed interactively (unless significant and skilled programming effort is applied). To conclude, PyPedia is not a tool for interactive analysis per se but a code repository that helps other tools to perform interactive analysis.

### Future work

Our first priority in the future is to submit additional articles as simple PyPedia users. To enhance the software quality we plan to introduce a voting mechanism through which the transition of articles from the User to the Validated category will be more transparent and objective (for PyPedia installations using this mechanism).

Moreover we plan to support execution of computational intensive PyPedia methods through remotely submitting jobs to cluster environments via the SSH interface. A similar future step is to build execution environments ‘on-the-fly’ in the cloud (i.e. Amazon EC2). To do that we plan to add additional parameters that will determine the system architecture, the CPU and the memory requirement of the methods. The users can submit their cloud credentials and the PyPedia environment will setup the environment, submit the computational task, fetch the results and release the resources.

In order to improve the uniformity of the methods we plan to experiment with extensions that offer semantic integration [52]. The naming of the articles and the parameters of the methods should follow the same schema and new content should be forced to adhere these directions. For example parameters that represent a nucleotide sequence in FASTA format should have the same name across all PyPedia methods. In Wikipedia, articles that belong to the same semantic category contain a uniform structure. Similarly PyPedia can aim to standardize bioinformatics methods.

Furthermore we believe that open and editable code is one of the two fundamental components of modern science. The other is open and easy accessible data [53, 54]. Packages likes BioPython and PyCogent include methods to query online repositories and transfer data. Yet, a comprehensive list of data repositories in bioinformatics along with suitable access methods is still missing. For these reasons, we plan to catalogue these open repositories and develop methods to streamline the transfer and management of large scientific data.

## Conclusions

PyPedia can be considered part of a family of e-science tools that try to integrate and connect all stakeholders involved in a bioinformatics community [26, 29, 55]. Therefore special care has been given to provide interfaces to ease the integration with external via RESTful web services [56, 57], programming APIs, online method execution and traditional HTML forms. With this, PyPedia can be useful as central method repository for a bioinformatics project, laboratory or multi-center consortium. In addition, PyPedia can be also conceived as an experimentation platform where users can test and evaluate methods, try various parameters and assess the results. To evaluate PyPedia we presented the concept at several conferences: Bioinformatics Open Source Conference (BOSC 2012), EuroPython 2012 and EuroSciPy 2012 Below we summarize positive and negative criticisms received to the concepts described above.

PyPedia attempts to address issues facing individual bioinformaticians and teams by offering an environment that promotes openness and reproducibility. Starting from experimentation users can generate initial results and ideas that they can share. Then they can create a draft article, add documentation and an HTML submission form and make the article appealing for other users to collaborate and improve it. From this they can offer and use the dependency-free version of their solution to other tools and environments for ‘real-world’ execution as part of daily business. The overhead of installation and configuration has been minimized whereas the User Interaction is familiar to any Wikipedia user.

The programming language of the content methods is Python and was chosen for the simplicity, readability and the dynamic that exhibits in the bioinformatics community. Python has been characterized as a ‘glue language’, meaning that is suitable for integrating heterogeneous applications in a simple and intuitive way that was confirmed in this pilot.

We provide PyPedia as open source solution for any individual or group to adopt, to use as sharing system or to publish methods as supplement to a paper. Meanwhile we plan to keep maintaining the public pilot site so that it may evolve in a more broadly used method catalogue. Although PyPedia has been developed with the particular needs of the bioinformatics software community in mind, we believe that the same design principles can benefit other research domains. Consequently, we plan to embrace content coming from other scientific disciplines.

## Availability and requirements

**Project name:** PyPedia**Project home page:**http://www.pypedia.com**Operating system(s):** Platform independent**Programming language:** Python**Other requirements: Anaconda:**https://www.continuum.io/downloads**License:** BSD 2-Clause License

## Additional files

Additional file 1
**Additional documentation and technical manual of PyPedia.** (DOCX 135 kb)

Additional file 2
**Detailed discussion and presentation of different user groups and articles categories as well as some security issues.** (DOCX 134 kb)

Additional file 3
**A scenario of bioinformatics analysis with existing articles in pypedia.com through IPython.** (PDF 107 kb)

## Abbreviations

API: Application Programming Interface
CPU: Central Processing Unit
GWAS: Genome-Wide Association Study
HTTP: HyperText Transfer Protocol
SNP: Single-Nucleotide Polymorphism
VCF: Variant Call Format
XML: Extensible Markup Language
