# Supplementary material for: PyPedia: using the wiki paradigm as crowd sourcing environment for bioinformatics protocols
Source: Source Code Biol Med. 2015 Nov 19;10:14. doi: 10.1186/s13029-015-0042-6 (PMC4652372; doi:10.1186/s13029-015-0042-6)
Supplement: Additional file 1 — Additional documentation and technical manual of PyPedia. (DOCX 135 kb) [file 13029_2015_42_MOESM1_ESM.docx]

Supplementary file 1

Additional documentation of pypedia.com

An online and frequently updated documentation page for [www.pypedia.com](http://www.pypedia.com) exists here: <http://www.pypedia.com/index.php/PyPedia:Documentation>.

Here we present the main functionality of pypedia as of December 2014

## Basic functionality

PyPedia is a normal installation of MediaWiki, the Content Management System that Wikipedia is based on. Additionally some Hooks have been written in order to alter the normal behavior of MediaWiki when it creates a new article and when it saves an edit. The new behavior allows only for a specific structure for each article. In each article an author describes the documentation, the parameters, the source code and the unit tests of a python method. This method has the same name as the article. Once this article is saved, the code, can be called by a simple function call (no import needed) by another PyPedia article or loaded locally by using the PyPedia python library. As with Wikipedia, every edit is instantly accessible to all users.

## Regarding safety

See also: Supplementary file 2

Articles can belong in one of two virtual namespaces.

The articles ending with "_user_<username>" are created and edited by PyPedia users who define the content and the permissions. These articles can have (almost) any kind of python code. The format of the title of these articles is: <Function or Class name>_user_<Username>. For example: Foo_user_JohnDoe. These articles belong to the _user_ virtual namespace.

The normal articles are articles taken from the _user_ virtual namespace that fulfill certain qualitative criteria. These criteria include: qualitative, "pythonic", commented code that solves a known problem with complete documentation, parameters and unitests. These articles can be edited only by the admins and shouldn't call any _user_ articles.

These virtual namespaces shouldn’t be confused with the mediawiki namespaces. All the articles belong to the same main mediawiki namespace. The difference between _user_ and normal articles is the naming of the title. With this distinction we allow users to experiment and define their own sub-communities in PyPedia while there is a space for objectively qualitative and safe articles in the normal virtual namespace. A user can request the move of a _user_ article to the Validated category by making a comment the Talk pages. There are thoughts to include a voting mechanism in the future.

This practically means that articles in the _user_ virtual namespace shouldn't be considered safe. Users shouldn't run these articles in their local computer and the pypedia python library forbids this. Nevertheless, since the code is open for download there isn’t any mechanism that prevents users from running it. Subsequently users should be aware of the potential security risks that are taking. It is perfectly safe though to run these methods via the "Execute in browser" button in each article (or through the Main Page). This is because through the "Execute in browser" button, the code is run in a custom python sandbox and not in user’s local computer. Only what is printed in the output (or the errors) is printed in the user’s browser.

Moreover, it is safe to run methods that belong in the normal namespace (that is articles not containing the _user_ part, i.e. Hanoi towers). These articles have been moved there after thorough testing of their validity and safety. Of course a _user_ article is never called by an article in the normal namespace. As with every open source project, no guarantee is given for the security, validity and efficiency of the provided code.

## User roles

Anonymous Users can only edit the Talk Pages and the Development Code section of each article. The code in the Development Code section is not parsed, run or downloaded. It is there to allow anyone to make corrections or suggestions to the authors of an article.

Signed in users can only create articles with the name: <Function or class name>_user_<Username>. The creator of an article is the only one allowed to edit this article. Users can change the permissions of each section of their articles and allow other users to edit specific sections by adding other user's usernames in the permissions sections. For example if we want to allow JohnDoe to edit the Documentation section of an article we should add "JohnDoe" with a comma next to our username in the Documentation Permissions section.

Admins can edit the normal virtual namespace. They can also delete or alter a User article if it is deliberately harmful, or misleading.

## License

The license of all the content is the Simplified BSD License: <http://www.pypedia.com/index.php/PyPedia:License>

## Editing the parameters of an article

A user can define the parameters of a method. The description uses a simple XML schema that is similar to the one used by the Galaxy Tool. The whole Parameters sections have to be included in a <inputs></inputs> tag. In these tags you can define:

<param

name="<name of the parameter>"

type="<data or eval>"

value="<the pre-inserted value in the textbox">

label="<The text displayed before the textbox>"/>

- Text Boxes:
  - name is the name of the parameter. Use the same name as an argument in the function defined in this article in order to access the value of the parameter.
  - type could be "data" for alphanumeric values, or eval for any python expression. This distinction happens on order to wrap with double quotes any alphanumeric constant.
  - value the pre-inserted value in the textbox
  - label the text displayed before the textbox.

<param

name="name of the parameter"

type="select"

label="The text displayed before this combobox"

<option value="Name_of_first_value">first_option</option>

<option value="Name_of_second_value">second_option</option>

</param>

- Combo Boxes:
- name of the name of the parameter. Use the same name as an argument in the function defined in this article in order to access the value of the parameter.
- type it should be "select"
- label the text displayed before the combo box

## The Code

In this section exists the code of the article. The code should be any Python 2.7 script. For example:

<source lang="py">

def Foo_user_JohnDoe(arg_1 = None, arg_2 = None):

print "function foo"

print "Arguments:", arg_1, arg_2

return 42

</source>

Some notes:

- The source tags are needed.
- The name of the function (or class) should be the same as the title of the article.
- The names of the arguments in the function should have the same name as the name of the parameters.
- Calling other methods: Simply call a method in an article by writing a function call: ArticleName(argument_Lists)
- Synonymous methods can be defined by creating redirect articles. For example the article: Hoo_user_JohnDoe could be a redirect to the article Foo_user_JohnDoe. To create a redirect substitute all the text of the Hoo_user_JohnDoe article with the text: #REDIRECT [[Foo_user_JohnDoe]]

## Unitests

The Unitests sections contain functions that act as assertions for the correction of the code. These functions should return True/False values according to the validity of the test. After an edit of the code, all unitests functions are evaluated. If any unitest fail then the edit is not saved. Unit tests are functions that have any name, no arguments and return True or False values.

An example of a unit test to verify that a function returns the expected value might be:

Alternatively, you can return a string that gives more details why the Unit test failed:

def unitTest():

return Foo_user_JohnDoe() == 42

def unitTest():

if Foo_user_JohnDoe() != 42:

return "This edit broke the function Foo_User_JohnDoe. It didn't return the correct value (42)"

return True

## Permissions

Here a user can define permissions for specific section edits. Each section can be edited only by the list of users explicitly defined here. The list should be coma separated. Initially only the creator of the page is allowed to edit all sections. Special user permissions are:

- "ALL" : All users (even anonymous) are allowed to edit this section.
- "SIGNED": All signed in users are allowed to edit this section.

## "Download Code" button

In each article, a user, can fill in the parameters of an algorithm. Then she can click the "Download Code" button and a <Article_name>.py file is downloaded (for example Hello_world.py). This file contains all the necessary python code needed to run the method. To execute the code type:

In this script both the code, the documentation and the parameters are included.

> python <Article_name>.py

## Execute on browser button

With the "Execute on browser” button the same code that is downloaded with the "Download code" button is sent to a custom python sandbox. The code is executed and the results are shown back in the browser.

The python that is installed in the sandbox is the anaconda that supports many scientific packages: https://store.continuum.io/cshop/anaconda/

## "Execute on remote computer" button

There is also the ability to let PyPedia login via SSH in a remote computer, execute the code and fetch the results. A user can simply fill in the parameters in the form of each article and press the execute button. The user have to be logged in and she has to have declared the hostname, username and the path where she wants the computation to take place. To declare these elements, a user has to edit her user page (for example: http://www.pypedia.com/index.php/User:JohnDoe) and add the following section at the end of the page:

==ssh==

host=www.example.com

username=JohnDoe

path=/home/JohnDoe/runPyPedia

This text is not saved in the wiki. The User table of the mediawiki has been altered in order to contain four more columns: The host, the username, the port and the path of a remote computer. A user also needs to have installed the PyPedia python library and the ssh_pyp_client utility in the remote computer. In order to do that, go to the execution path of the remote computer (in our case /home/JohnDoe/runPyPedia) and run:

git clone git://github.com/kantale/pypedia.git

wget https://raw.github.com/kantale/PyPedia_server/master/utils/ssh_pyp_client.py

Now when a user presses the "Execute on remote computer" button. A text appears that asks for the password of the user’s remote computer. The user the fills in the password and presses the "GO" button. The code then is executed in the remote code and three values are shown. (1) What the method printed in standard output, (2) What the method printed in standard error and (3) what the method returned. The output is shown in the current page and it looks like this:

Error:

...

Printed:

...

Returned:

...

Notes:

- The username declared in the ==ssh== section doesn't have to be the same with the PyPedia username
- A user can run any PyPedia method with this way, regardless if she has editing rights or not.
- The connection will close if the method does more than 30 seconds.
- When running in a remote computer, PyPedia uses the python that is declared in the $PATH variable of the remote computer. Since this run through ssh connection the $PATH should be declared in .bashrc since it is sourced by non-interactive non-login shells.

## PyPedia python library

To execute the PyPedia code locally we need a special library that connects to www.pypedia.com and downloads and manages the code. To install the library:

git clone git://github.com/kantale/pypedia.git

After installing the pypedia library simply type (assuming a suitable python environment):

The first statement (import pypedia), maintains a connection to the wiki while the second (from pypedia import Hello_world) downloads and imports the function or class Hello_world. Of course pypedia needs to be imported once, whereas for any function that we want to import we need a "from pypedia import XXXX" statement. We only have to import the main function that we want to run: If the function foo() calls goo() and we don't want to call goo() explicitly then we only have to import foo(). The docstring of the function or class contains the documentation that exists in the Documentation section of the article. For example try: print Hello_world.__doc__

>>> import pypedia

>>> from pypedia import Hello_world

>>> Hello_world()

Hello World!

>>>

Additional options:

- pypedia.enable_cache = True , to download articles only if there aren't already downloaded. To clear the cache delete everything in the directory pypedia/pypCode/
- pypedia.debug = True , for debug info
- pypedia.warnings = False , to suppress warnings
- pypedia.before_timestamp. Import the most recent revision of the articles right BEFORE the timestamp that you set in this variable. The format should be "YYYYMMDDHHMMSS". For example the following code will import the last revision of the method Hello_world as it was before the 16/4/2012.

>>> import pypedia

>>> pypedia.before_timestamp = "20120416000000"

>>> from pypedia import Hello_world

This revision filter is applied recursively to all functions and classes that are also imported. A user should use this option to reproduce an analysis that was done by pypedia at a specific time ignoring all the subsequent changes done to the articles.

## Editing articles locally

In general, editing of the articles can happen through the www.pypedia.com website. By clicking three times in the mediawiki editing textbox, it is converted to a (relatively) code-friendly environment. There is also the option to add and edit an article locally through the PyPedia python library.

**Attention**: by default the library connects to pypedia by using a preset account with username: pypediauser and password: pypediauserpw. This account does not have any edit privileges. When we are trying to edit an article with the pypedia library, we should make sure to change this with our own account username and password. To set a username and password:

Alternatively (and mainly for security reasons) we can create the file: ".pyp" in our home directory. And set these values:

pypedia.username = "JohnDoe"

pypedia.password = "secretpassword"

Make sure that the file has 600 permissions:

#> chmod 600 ~/.pyp

username = "JohnDoe"

password = "secretpassword"

After setting these values, to create a new article we can use the "add" function. For example:

import pypedia

pypedia.add("Foo_user_JohnDoe")

This method will create the article Foo_user_JohnDoe in pypedia.com. Subsequently we can edit the file : pypedia/pyp_code/pyp_Foo_user_JohnDoe.py with our favorite code editor. To "push" the results to pypedia.com we can use the push function:

import pypedia

pypedia.push()

This method will identify all the files that have been edited locally and it will try to upload them to pypedia.com . To upload a single file use:

If a file has been edited locally and the changes have not been pushed to pypedia.com then we can not download this article locally unless we set the pypedia.force_imports = True variable.

pypedia.push("Foo_user_JohnDoe")

## Forking an article

Forking is the procedure of creating a personal copy of the current version of an article. By pressing the button "Fork this article" on the top of any article, a new article is created that contains almost the same content. The name of the article and the permissions are changed in order to comply with the new owner. We cannot fork an article if we are not signed in or if the article to be created already exists. We can fork an article regardless if it belongs in the User or in the Validated category. If we fork a Validated article then the created article will belong in the User category (see also Supplementary file 2).

## The REST interface

PyPedia offers a simple REST interface to access the constraint-free, standalone version of articles. The constraint-free version of an article contains the source code of the article plus the source code of the articles that depends on. For example if Foo() calls Goo() and you request the constraint-free version of Foo() then the source code of both Foo and Goo will be delivered. We can also use the standard REST api of MediaWiki for more extensive queries.

### The get_code command

The format of the command is:

http://www.pypedia.com/index.php?get_code=<python code>

<python code> can contain any python code that depends on functions / classes that are hosted in PyPedia articles. The returned content is the code hosted in PyPedia that is required by the <python code> in order to run without any further requirements (except of course if the articles require external packages i.e. numpy that we should have locally installed). The content returned is in plain text format. It might appear that the indentation is lost but this is because this content is not designed for browser view. To view the code with indentation, check the source of the page from the browser.

Example:

- Note: If you copy the above to your browser URL bar, the browser will convert the special characters to URL suitable values.

http://www.pypedia.com/index.php?get_code=Pairwise_linkage_disequilibrium([("A","A"), ("A","G"), ("G","G"), ("G","A")], [("A","A"), ("A","G"), ("G","G"), ("A","A")])

A very convenient way to use this is through the wget / curl commands. For example any of the following commands:

or

wget -O code.py 'http://www.pypedia.com/index.php?get_code=print Pairwise_linkage_disequilibrium([("A","A"), ("A","G"), ("G","G"), ("G","A")], [("A","A"), ("A","G"), ("G","G"), ("A","A")])'

will create the file code.py with all the required code to execute the command in the URL. Run: python code.py to see the execution results.

curl --data-urlencode 'get_code=print Pairwise_linkage_disequilibrium([("A","A"), ("A","G"), ("G","G"), ("G","A")], [("A","A"), ("A","G"), ("G","G"), ("A","A")])' http://www.pypedia.com/index.php --output code.py

### The dl_code command

The format of the command is:

http://www.pypedia.com/index.php?dl_code=_=<python function call>

For example:

http://www.pypedia.com/index.php?dl_code=_=Pairwise_linkage_disequilibrium([("A","A"), ("A","G"), ("G","G"), ("G","A")], [("A","A"), ("A","G"), ("G","G"), ("A","A")])

This command will take the contraint-free code necessary for executing the function call and save it to the file <Function>.py . In this file, the returned value of the function is saved to the _ variable. The file will be downloaded by the browser.

### The run_code command

http://www.pypedia.com/index.php?run_code=<python code>

The format of the command is:

For example:

http://www.pypedia.com/index.php?run_code=Hello_world()

The command will execute the submitted python code and will fetch the results of the standard output. In case the output has more than 500 lines only the **last** 500 will be printed.

### The b_timestamp parameter

The **get_code**, **dl_code** and **run_code** commands can be combined with the **b_timestamp** parameter that defines the date/time that we want to retrieve the revision from. The value of **b_timestamp** does not have to be one of the specific timestamps of an article. In that case, the version of the articles that are retrieved is the most recent version that is right **BEFORE** the timestamp declared. The format of the parameter is YYYYMMDDHHMMSS. For example assume that the article Foo contains three revisions timestamped 20120101000000, 20120102000000, 20120103000000 (1st, 2nd, 3rd of January 2012 respectively).

- If we provide the value 20110101000000 to b_timestamp (before the 1st revision) then the 1st revision will be retrieved.
- If we provide the value 20120101100000 to b_timestamp (after the 1st and before the 2nd) then the 1st revision will be retrieved.
- If we provide the value 20120102000000 to b_timestamp (exactly the same as the 2nd) then the 2nd revision will be retrieved.
- If we provide the value 20120104000000 to b_timestamp (after the 3rd revision) then the 3rd revision will be retrieved.

This filter is applied to all the articles that Foo invokes. An example of using this parameter is:

http://www.pypedia.com/index.php?b_timestamp=20120105113000&get_code=Hello_world()

The timestamps of the revisions of the articles (in the "view history" link) does not appear in the presented format and may have a difference from current time. To have a uniform view between the timestamps in the b_timestamp parameter and the timestamp in the "view history" page, we should do the following: Go to My preferences --> Data and Time. Select the last option (something like 2012-04-18T12:43:02) from the various time formats. From the time zone select "specify offset" and then fill "00:00". By doing that the time appeared in the "view history pages" will have the format 2012-04-18T12:43:02 which is easy to convert to 20120418124302 that is the format used by the **b_timestamp** parameter.

## Using the REST interface to share and reproduce an analysis

The commands and parameters described above provide an easy interface to share the complete analysis that is done with functions and classes hosted in PyPedia. The main purpose of sharing the analysis is to be able to reproduce it and archive it. Suppose that we have generated some results by using the function in article Foo:

def Foo(input_filename):

return Apply_analysis(input_filename)

The function takes as input a filename and returns a float value. Since the methods are prone to change (if for example we are using methods that other users can edit), we mark the timestamp (say 20120102101010) when the methods had the desired behavior. Then we can share the analysis by sharing the following URL:

Anyone can use either the curl or the wget tool to save the code that is returned by this url in a python file and then execute it. If we cannot use curl or wget we can share the following URL:

http://www.pypedia.com/index.php?b_timestamp=20120102101010&dl_code=_=Foo("input_file.txt")%0Aprint _

http://www.pypedia.com/index.php?b_timestamp=20120102101010&get_code=print Foo("input_file.txt")

By applying this URL in a browser, a file "Foo.py" is downloaded that includes the analysis code and the code to print the results. Still we will have to share the data file: "input_file.txt". The sharing of data files is currently left to users. To circumvent this and to minimize the effort spent to reproduce an analysis, we encourage users, to create functions that fetch data from public repositories or/and from other urls. The above urls can also be used for archiving.
