# Supplementary material for: PyPedia: using the wiki paradigm as crowd sourcing environment for bioinformatics protocols
Source: Source Code Biol Med. 2015 Nov 19;10:14. doi: 10.1186/s13029-015-0042-6 (PMC4652372; doi:10.1186/s13029-015-0042-6)
Supplement: Additional file 2 — Detailed discussion and presentation of different user groups and articles categories as well as some security issues. (DOCX 134 kb) [file 13029_2015_42_MOESM2_ESM.docx]

Supplementary file 2

Security and Article distinction in pypedia.com

In a collaborative, open and freely available content management system, special measures have to be taken in order to prevent edits that are malicious, erroneous and in general non-contributing. In pypedia, the most important measure is the division of the articles into two categories, Validated and User. Articles that belong in the Validated category satisfy all the qualitative criteria of PyPedia. These criteria are:

1. The method should solve a previously defined and known bioinformatics problem.
2. The code should be concise, easy to understand, commented and “pythonic”.
3. The method should have unit tests that cover most of the method’s functionality.
4. The documentation should be complete, extensive, and precise with references and examples for common cases.
5. The article should contain a well-formed parameters section.

These criteria are generic and are constantly under refinement in order to satisfy the needs of a dynamic and growing community. The Validated category is in contrast with the User category that contains articles created by arbitrary users. In the User category, users are allowed to submit any content without any active restrictions and quality criteria. The administrators’ intermissions are very limited and happen only in cases of deliberately malicious and misleading content. Beside that, we let the content flourish under the general philosophy of the “wiki” websites. Because of the general unsafe nature of the User methods, we allow their execution only in the python sandbox, where it is impossible to cause any harm. Users can still execute these methods in their local unprotected environment by explicitly setting a parameter in the PyPedia python library. Moreover, the methods in Validated category are not allowed to call methods in the User category whereas the opposite is allowed. The articles in the Validated category are created by carefully inspecting the pool of articles in the User category for content that satisfies the aforementioned quality criteria. In case a User article is qualitative enough we create a copy in the Validated category. With this approach, the community-driven development of the article can continue in the User category whereas the Validated category contains a stable, tested and approved version of the article. Users can suggest the moving of an article to the Validated category by requesting it in the “Discussion” page of the article.

The distinction between these categories is very simple and is based on the naming of the title of the articles. The title of the articles in the User category has the format: <MethodName>_User_<Username>, for example foo_User_JohnDoe, which can be interpreted as the method “foo” created by the user JohnDoe. The same article in the Validated category would have the title “foo”. The advantage of this distinction is that many users can have a method with the same name, preventing naming “hijacking”. Moreover this allows the creation of mutually trusted sub-communities and sub-namespaces where the creators and the contributors of the articles are easily identified.

If a user wants to edit an article but she does not have the appropriate permissions she can press the “Fork this article” button at the top of the article. This creates a copy of the article that is owned by this user, meaning that she can edit the article and define its edit permissions. The new article has an updated name according to the naming schema presented before. “Forking” is a technique also used in the known social coding platform, github.

To facilitate these operations, users are divided into three privilege categories. The administrators check for deliberate malicious edits, promote articles from the User to Validated category and in general perform “housekeeping” tasks. Signed in users are allowed to create articles according to the presented naming scheme and edit the sections that have permission to. Finally, anonymous are allowed to edit the “Development Code” sections and the Talk pages of the articles.
