# Supplementary material for: PyPedia: using the wiki paradigm as crowd sourcing environment for bioinformatics protocols
Source: Source Code Biol Med. 2015 Nov 19;10:14. doi: 10.1186/s13029-015-0042-6 (PMC4652372; doi:10.1186/s13029-015-0042-6)
Supplement: Additional file 3 — A scenario of bioinformatics analysis with existing articles in pypedia.com through IPython. (PDF 107 kb) [file 13029_2015_42_MOESM3_ESM.pdf]

Additional File 3. A scenario of bioinformatics analysis with existing articles of pypedia.com

In this notebook we demonstrate how pypedia can be used to perform simple bioinformatics analysis with openly available data. We also demonstrate how IPython can be combined with pypedia.com. IPython notebook is a convenient tool not only for performimg an analysis but also to distribute and reproduce it. This file is a PDF conversion of the IPython notebook file that contains the complete analysis and can be downloaded from this link: https://gist.github.com/kantale/e09e43ce7aac69e2015a. To reproduce the analysis simply load this file to a local IPython notebook.

1 Prerequisites

Initially we assume that the pypedia library has been installed locally. To do that run the following from the computer's terminal (not in python):

```
git clone git://github.com/kantale/pypedia.git
```

This example assumes a python 2.7 (or higher but not 3.x) environment. The first command imports the pypedia library. This library maintains a connection between the local environment and pypedia.com

```
In [1]: import pypedia
```

2 Downloading a dataset

For this demonstration we can experiment with the files available in: http://pngu.mgh.harvard.edu/~purcell/plink/res.shtml For example the file that contains the CEU founders (release 23, 60 individuals, filtered 2.3 million SNPs) can be downloaded locally with the following commands.

```
In [2]: from pypedia import download_link
        link = 'http://pngu.mgh.harvard.edu/~purcell/plink/dist/hapmap_CEU_r23a_filtered.zip'
        download_link(link)
```

```
Extracted filename from url: hapmap_CEU_r23a_filtered.zip
Saving at: hapmap_CEU_r23a_filtered.zip
Downloading: hapmap_CEU_r23a_filtered.zip Bytes: 31287683
[****************100%******************]  31285249 of 31287683 complete
```

Next step is to unzip the downloaded file

```
In [3]: from pypedia import unzip
        unzip('hapmap_CEU_r23a_filtered.zip', './')
```

```
Unzipping file: ./hapmap_CEU_r23a_filtered.bed
Unzipping file: ./hapmap_CEU_r23a_filtered.bim
Unzipping file: ./hapmap_CEU_r23a_filtered.fam
```

3 Reading a dataset

The data are in binary BED / BIM / FAM format. We can apply a reader to this data. Readers are python generators.

```
In [4]: from pypedia import BED_PLINK_reader
        reader = BED_PLINK_reader(
                  'hapmap_CEU_r23a_filtered.bed',
                  'hapmap_CEU_r23a_filtered.bim',
                  'hapmap_CEU_r23a_filtered.fam')
```

As we can see from the documentation (http://www.pypedia.com/index.php/BED_PLINK_reader), BED_PLINK_reader is a python generator. The first item that it generates is a dictionary that contains the family, sample and phenotype information of the dataset:

```
In [5]: header = reader.next()
```

```
Loading: hapmap_CEU_r23a_filtered.bim
Read 2333521 SNPs
Loading: hapmap_CEU_r23a_filtered.fam
Read 60 Individuals
```

The subsequent items of this generator contain genotype information. Suppose that we are interested on two particular SNPs: rs16839450 and rs16839451. We can extract the genotypes of these snps:

```
In [6]: interesting_snps = [x for x in reader if x['rs_id'] in ['rs16839450', 'rs16839451']]
```

```
BED file type: SNP_major
[****************100%******************]  2330001 of 2333521 complete
```

4 Basic genetics statistics

One experiment we can perform is to check if the SNPs that we selected are in LD (Linkage Disequilibrium):

```
In [7]: from pypedia import Pairwise_linkage_disequilibrium as PLD
        ld = PLD(interesting_snps[0]['genotypes'], interesting_snps[1]['genotypes'])
        ld
```

```
Out[7]: {'Dprime': 1.0,
         'R_sq': 0.8896046482253379,
         'haplotypes': [('CA', 0.7807017543859649, 0.5986842105263158),
          ('CG', 0.00877192982456141, 0.19078947368421056),
          ('TA', 6.268997962918469e-18, 0.15964912280701754),
          ('TG', 0.21052631578947367, 0.050877192982456146)]}
```

The result ('R_sq' ~= 0.89) shows that these SNPs are in LD.

Subsequently we can measure the Hardy Weinberg Equilibrium of all SNPs:

```
In [8]: reader = BED_PLINK_reader(
                  'hapmap_CEU_r23a_filtered.bed',
                  'hapmap_CEU_r23a_filtered.bim',
                  'hapmap_CEU_r23a_filtered.fam')
        header = reader.next()

        from pypedia import hardy_weinberg_equilibrium as hwe
        hwe_values = [hwe(x['genotypes']) for x in reader]
```

```
Loading: hapmap_CEU_r23a_filtered.bim
Read 2333521 SNPs
Loading: hapmap_CEU_r23a_filtered.fam
Read 60 Individuals
BED file type: SNP_major
[***************100%*****************]  2330001 of 2333521 complete
```

We can also create a Q-Q plot that will show if the p-values are normally distributed, or if an inflation is taking place:

```
In [9]: %matplotlib inline
```

```
In [10]: from pypedia import qq_plot
         qq_plot(hwe_values, show_plot='on_screen')
```

```
lambda: 0.483347
```

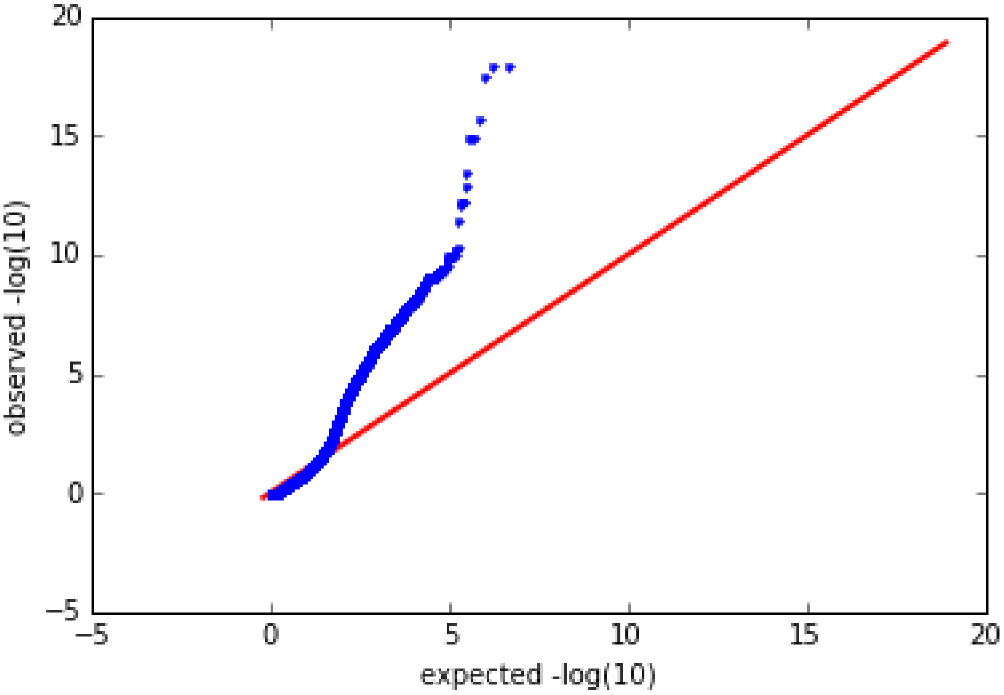


```
Out[10]: <module 'matplotlib.pyplot' from '/Users/alexandroskanterakis/anaconda/lib/python2.7/site-packa
```

There is clearly an inlfation of the hardy weinberg statistic in this dataset.

Finally we can also perform a genetic association test. For demonstration purposes we can assign random values as phenotypes:

```
In [11]: #Get the number of samples
         samples = len(header['sex_ids'])

         #Split cases and controls equally
         controls = samples/2
         cases = samples - controls
```

```
#Create random phenotype
pheno = [1] * cases + [2] * controls
from random import shuffle
shuffle(pheno)
```

The genetic_association_test method contains a collection of genetic association tests.

```
In [12]: from pypedia import genetic_association_test as gat
```

We can create a more complex analysis, for example report all SNPs where they pass the hardy weinberg statistic (p-value>0.0001) and the p-value of the genotypic test for association with the phenotype is lower than 10ˆ-4. Of course, since this is a random assigned phenotype we expect the method to find a few SNPs.

```
In [13]: # "rewind" reader
         reader = BED_PLINK_reader(
                  'hapmap_CEU_r23a_filtered.bed',
                  'hapmap_CEU_r23a_filtered.bim',
                  'hapmap_CEU_r23a_filtered.fam')
         header = reader.next()

         reported_snps = []

         #Iterate through all markers
         for marker in reader:
                  #Get the genotype
                  genotypes = marker['genotypes']

                   #Discard markers with low hardy weinberg test values
                  hwe_value = hwe(genotypes)
                  if hwe_value < 1e-4:
                          continue

                   #On the remaining snps perform an association test
                  assoc = gat(genotypes, pheno, tests=['GENO'])
                  p_value = assoc['GENO']['P']

                   #Report SNPs with p_values < 10^-4
                 if p_value < 1e-4:
                          reported_snps += [(marker['rs_id'], str(p_value))]

         #Print first 10 reported SNPs
         for s in reported_snps[0:10]:
             print 'SNP: %s  p_value: %s' % (s[0], s[1])
```

```
Loading: hapmap_CEU_r23a_filtered.bim
Read 2333521 SNPs
Loading: hapmap_CEU_r23a_filtered.fam
Read 60 Individuals
BED file type: SNP_major
[***************100%*****************]  2330001 of 2333521 complete SNP: rs9729887  p_value: 7.4171563[illegible]
SNP: rs4650608  p_value: 4.25942698892e-05
SNP: rs12120254  p_value: 5.56259796442e-05
SNP: rs12022730  p_value: 8.07413948606e-05
SNP: rs3806412  p_value: 5.29801197352e-05
```

```
SNP: rs6693754  p_value: 6.08087642015e-05
SNP: rs17380246  p_value: 5.9213847799e-05
SNP: rs17371791  p_value: 6.72423732165e-05
SNP: rs11124566  p_value: 8.67876069542e-05
SNP: rs2540973  p_value: 4.51223345855e-05
```

5 Saving a method in pypedia.com

Suppose that we have developed a function (or class) and we want to make it part of the pypedia collection. We can create and edit an article in the pypedia.com website as with any other wiki. Alternativelly we can use the pypedia library to complete this task. To do that we should already have an account in pypedia.com. Then we must declare our username and password locally:

```
In [2]: pypedia.username='JohnDoe'
        pypedia.password='secretPassword'
```

Then we can create a new article in pypedia.com by using our account. Some notes:

- It is very important the name of the article (and the name of the function or class) to end in "user_< Username >". (See supplementary file 2)
- The first letter of the username should be in capital.
- In the documentation page we describe how to universally define these values (for security purposes).

For example:

```
In [3]: pypedia.add('foo_user_JohnDoe')
```

```
Article foo_user_JohnDoe saved
Next:
  Edit the article online: http://www.pypedia.com/index.php/foo_user_JohnDoe
  Or edit the article locally: /Users/alexandroskanterakis/del/pypedia/pypCode/pyp_foo_user_JohnDoe.py
    To push the changes to pypedia.com run: pypedia.push()
```

As it is indicated in the output messages there two ways to continue. Either to edit the article online (http://www.pypedia.com/index.php/foo_user_JohnDoe) or open the the file pyp_foo_user_JohnDoe.py with your favorite text editor, add the desired functionality and then type:

```
In [4]: pypedia.push()
```

6 Additional information

Additional documentation and functionality of the pypedia library can be found here: http://www.pypedia.com/index.php/PyPedia:Documentation.
